# Auditory Affective Norms for German: Testing the Influence of Depression and Anxiety on Valence and Arousal Ratings

**DOI:** 10.1371/journal.pone.0030086

**Published:** 2012-01-19

**Authors:** Philipp Kanske, Sonja A. Kotz

**Affiliations:** 1 Neurocognition of Rhythm in Communication Group, Max Planck Institute for Human Cognitive and Brain Sciences, Leipzig, Germany; 2 Department of Cognitive and Clinical Neuroscience, Central Institute of Mental Health, Mannheim, Germany; Federal University of Rio de Janeiro, Brazil

## Abstract

**Background:**

The study of emotional speech perception and emotional prosody necessitates stimuli with reliable affective norms. However, ratings may be affected by the participants' current emotional state as increased anxiety and depression have been shown to yield altered neural responding to emotional stimuli. Therefore, the present study had two aims, first to provide a database of emotional speech stimuli and second to probe the influence of depression and anxiety on the affective ratings.

**Methodology/Principal Findings:**

We selected 120 words from the Leipzig Affective Norms for German database (LANG), which includes visual ratings of positive, negative, and neutral word stimuli. These words were spoken by a male and a female native speaker of German with the respective emotional prosody, creating a total set of 240 auditory emotional stimuli. The recordings were rated again by an independent sample of subjects for valence and arousal, yielding groups of highly arousing negative or positive stimuli and neutral stimuli low in arousal. These ratings were correlated with participants' emotional state measured with the Depression Anxiety Stress Scales (DASS). Higher depression scores were related to more negative valence of negative and positive, but not neutral words. Anxiety scores correlated with increased arousal and more negative valence of negative words.

**Conclusions/Significance:**

These results underscore the importance of representatively distributed depression and anxiety scores in participants of affective rating studies. The LANG-audition database, which provides well-controlled, short-duration auditory word stimuli for the experimental investigation of emotional speech is available in Supporting Information S1.

## Introduction

The sound of a voice is greatly influenced by an individual's affective state. Emotions change physiological parameters such as heart rate, blood flow, and muscle tension, which in turn alter vocal production. Emotional arousal, for example, increases laryngeal tension and subglottal pressure, resulting in increased intensity. Scherer (2003; [1]) reviewed investigations on emotional prosody and found a characteristic pattern including increased mean pitch for anger and joy, for example. To infer others' affective states, it is highly adaptive to deduce these emotional signals from prosody. Humans seem to be able to do this quite rapidly (for an example using a mismatch-negativity paradigm see [2]. In a model depicting the neural processes and correlates underlying emotional speech comprehension, [3] describe a neural network consisting of the auditory cortex, superior temporal areas, as well as inferior and orbito-frontal cortex.

Nevertheless, the number of studies on the neural basis of emotional prosody processing is still limited. One of the reasons for this is the complex nature of auditory stimuli. In particular, their progression over time makes it difficult to fully control onset and offset of stimulus presentation, in contrast to visual stimuli, for which the timing is more easily managed. For a number of experimental paradigms, this poses a serious challenge. Most studies on the attentional effects of emotional stimuli, for example, present stimuli only for brief lengths of time, which is difficult or even impossible with some auditory stimuli such as spoken sentences [4]. Furthermore, studying emotional speech requires reliable affective norms to ensure that the stimuli truly elicit emotional responses.

While there are a number of databases with affective norms available for nonverbal interjections [5], for Serbian [6], for Polish [7], for Finnish [8], for Russian [9], and for Slovenian [10], the two German selections only give norms for pseudo-speech [11] and sentences [12], but not for shorter auditory speech stimuli, such as single words.

Thus, the first goal of the present study was to establish a database of auditory emotional word recordings with affective norms of prosody. To this end, we selected 120 words from the Leipzig Affective Norms for German database [13], which includes visual valence and arousal ratings (next to other lexical factors) for visual word stimuli. For these stimuli, we made auditory recordings of each word spoken by a male and a female actor with corresponding emotional prosody. These recordings were rated again in valence and arousal to narrow down the selection to the most salient stimuli and to cross-validate the visual affective norms.

The second goal of the study was to test if depression and anxiety influence affective ratings. The emotional state of participants has been shown to modulate the neural responses in the amygdala and other regions to affective stimuli such as faces [14], [15], [16], pictures [17], words [18], and speech [19]. This effect has been reported in patient populations and in subclinical variations of anxiety and depression. Affective normative data may, therefore, be influenced by the emotional states of the rating participants, even if participants with mental disorders are excluded. This could bias the results of subsequent studies using stimuli selected from these published databases if the samples differ in depression and anxiety. It can be assumed that random selection of participants ensures equally representative distributions of emotional conditions in different samples, but if depression and anxiety really do modulate affective ratings, it would be helpful, if depression and anxiety scores of the rating samples were reported along with databases to help subsequent studies to check for compatibility with their own samples. Therefore, we asked all participants to complete the Depression Anxiety Stress Scales (DASS, [20]) and correlated the individual scores with the valence and arousal ratings of the speech stimuli. According to the extended tripartite model of depression and anxiety [21], depression is characterized by increased negative and reduced positive affect. Anxiety, in contrast, is mainly characterized by increased arousal (see also [22], [23]). Thus, we expect to find correlations of depression with more negative and less positive valence ratings, while anxiety should correlate with arousal ratings.

## Materials and Methods

### Participants

All participants gave informed written consent. The study was approved by the local ethics committee of the University of Leipzig and was conducted according to the principles expressed in the Declaration of Helsinki. A sample of 30 native German speakers was recruited from the University of Leipzig. There were 16 female participants; mean age was 23.2 years (SD = 2.8). All participants were right-handed according to the Edinburgh Handedness Inventory [24], with a mean laterality quotient of 87.8 (SD 20.4). All participants reported normal or corrected-to-normal vision and normal hearing.

The Depression Anxiety Stress Scales (DASS, [20]) were completed by each participant to obtain individual scores in current depression and anxiety, and additionally in stress levels. The DASS have been developed specifically to distinguish between depression and anxiety. Reliability is adequate, ranging from .84 to .91 in nonclinical samples.

### Materials and procedures

From the 1000 German nouns in the LANG database [13], we selected a subset of 120 words that were prototypical according to the following categories: (1) negative and high arousing, (2) neutral and low arousing, and (3) positive and high arousing. The descriptive statistics are displayed in Table 1. Negative and positive words did not differ in arousal (*p*>.20), but differed significantly from neutral words. The three groups also differed significantly in valence (all *p*<.001), resulting in the typical quadratic relationship of valence and arousal (*r*_quad_ = .88, *p*<.001). There were no significant differences in concreteness (which has been shown to interact with word emotionality [25]), frequency of written usage, number of letters or number of syllables between the categories (all *p*>.30). Several auditory recordings of each word were made with the emotional expression corresponding to the word's emotional valence, i.e. positive words were spoken with a happy tone of voice, negative words with an angry voice. The speakers were two professional actors who were native speakers of German. One of the speakers was female (30 years), the other male (28 years). Recordings were made with Algorec 2.1 (Algorithmix GmbH, Waldshut-Tiengen, Germany) and the sound files were further processed in PRAAT (Institute of Phonetics Sciences, University of Amsterdam). Two versions of each positive, negative, and neutral word from each speaker were chosen for the rating study. In total, participants were presented with 480 different auditory stimuli. To control for differences in loudness, all stimuli were normalized in sound intensity to 75 dB SPL.

**Table 1:** Descriptive statistics of the LANG-Audition database: The means and standard deviations (in parentheses) of the auditory recordings and the respective norms taken from the LANG database in visual word presentation are given.

	negative	neutral	positive
example word	Krieg [*war*]	Dampf [*steam*]	Kuss [*kiss*]
*Normative data of the auditory recordings (LANG-Audition)*			
rated auditory valence	2.3 (0.2)	5.0 (0.1)	7.3 (0.2)
female raters	2.0 (0.5)	5.0 (0.4)	7.7 (0.6)
male raters	2.6 (0.8)	5.0 (0.4)	7.3 (0.6)
rated auditory arousal	7.4 (0.3)	2.4 (0.2)	6.8 (0.4)
female raters	7.6 (0.7)	2.4 (1.2)	6.8 (1.0)
male raters	7.3 (0.5)	2.5 (0.9)	6.4 (0.9)
duration	0.6 (0.1)	0.6 (0.1)	0.8 (0.2)
mean pitch	280.8 (58.4)	159.7 (22.7)	262.1 (52.2)
maximum pitch	361.6 (73.6)	191.9 (28.5)	365.3 (87.0)
minimum pitch	179.6 (54.3)	124.0 (23.5)	153.4 (34.7)
pitch SD	56.7 (23.0)	21.8 (7.4)	70.2 (27.1)
*Normative data taken from LANG (Kanske & Kotz, 2010)*			
rated visual valence	2.4 (0.4)	5.0 (0.1)	7.5 (0.3)
rated visual arousal	6.8 (0.5)	2.3 (0.3)	6.7 (0.6)
rated concreteness	6.3 (1.5)	6.0 (1.3)	6.3 (1.8)
word frequency	11.0 (1.9)	11.3 (1.9)	10.7 (2.2)
number of letters	5.5 (1.1)	5.9 (1.1)	5.7 (1.3)
number of syllables	1.7 (0.5)	1.8 (0.4)	1.7 (0.4)

For each measurement, participants came to the laboratory for one session during which they rated word valence (negative - neutral - positive) and arousal (high arousing - low arousing). The order of the tasks was counterbalanced. Ratings were done on 9-point Likert scales. For valence and arousal ratings, the Self-Assessment Manikins [26], [27] were used. The assignment of the scale endpoints to the left and right was counterbalanced across participants. The instructions required participants to evaluate each stimulus as a whole and not selectively focus on semantics or prosody only. While rating, participants were seated in a comfortable chair in a sound-attenuated room and wore headphones (Sennheiser HD 202). Stimuli were presented with ERTS (experimental run time system, Berisoft Cooperation, Frankfurt, Germany).

### Description of the LANG-Audition Database

The database contains 240 auditory recordings of German nouns. There are two recordings of each word, one by a male and one by a female speaker. Normative data on valence and arousal obtained from 30 participants who evaluated each recording is included, as well as the normative data from the LANG on visually rated valence, arousal, and concreteness. Furthermore, the database includes lexical characteristics (frequency of written occurrence taken from the Wortschatz Lexikon of the University of Leipzig (http://wortschatz.uni-leipzig.de/), word length in number of letters and number of syllables), acoustic parameters (mean, minimum, maximum pitch, and pitch variation (SD)), and duration of the recordings. The database is included in Supporting Information S1.

## Results

### General ratings

**Figure 1:**
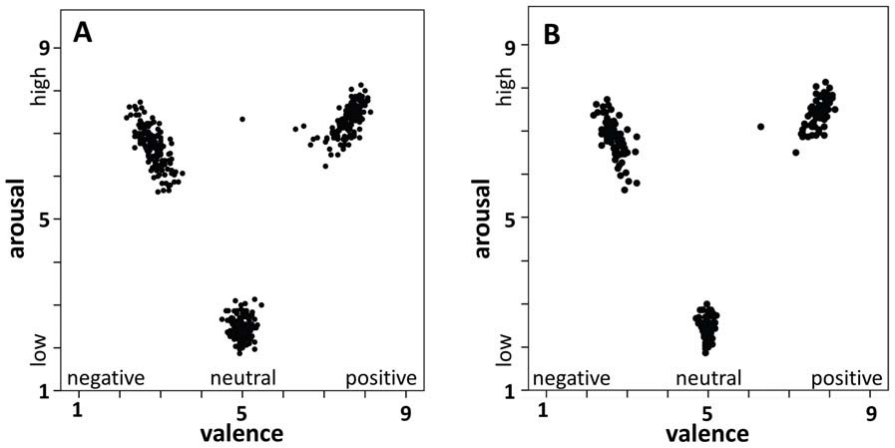
Scatter plots for valence and arousal ratings of the two versions of each of the 480 rated stimuli (A) and the 240 selected stimuli (B).

As can be seen in Figure 1A, almost all stimuli received distinct ratings in arousal and valence. From the two versions of each word, the one that was rated most unambiguously for one condition (e.g. least arousing and most neutral for the neutral word condition) was chosen (see Figure 1B). This resulted in 40 negative, 40 neutral, and 40 positive words, each spoken by the male and female speaker, resulting in 240 stimuli in total. The descriptive statistics for auditory valence and arousal ratings are displayed in Table 1. A one-way ANOVA yielded significant main effects of word group on valence (*F*(2,237) = 12810.0, *p*<.001) and arousal ratings (*F*(2,237) = 5167.6, *p*<.001). Repeated contrasts indicated that valence ratings of all three conditions differed significantly (negative<neutral<positive, all *p*<.001). Positive and negative words also differed from neutral words in arousal (*p*<.001). Interestingly, even though the arousal ratings of the visually presented negative and positive words did not differ, the negative auditory words were slightly more arousing than the positive words (*p*<.001). Both positive and negative words were significantly more arousing than neutral words (all *p*<.001). When correlating valence and arousal, we observed a quadratic relationship (*r*_quad_ = .98, *p*<.001), demonstrating the typical distribution of valence and arousal values comparable to visual word stimuli.

Naturally, the conditions also differed with respect to some of the acoustic parameters that constitute emotional prosody (see [1] for a detailed discussion of these characteristics; note, however, that stimuli were normalized in intensity, thus loudness means are not reported; duration: *F*(2,237) = 46.9, *p*<.001; mean pitch: *F*(2,237) = 153.3, *p*<.001; maximum pitch: *F*(2,237) = 170.7, *p*<.001; minimum pitch: *F*(2,237) = 39.4, *p*<.001; pitch variation (SD): *F*(2,237) = 114.0, *p*<.001). Recordings of positive words were significantly longer than those of neutral and negative words (*p*<.001). Regarding pitch, we found the following differences, for mean pitch: neutral<positive<negative, for maximum pitch: neutral<positive = negative, for minimum pitch: neutral<positive<negative, for pitch variation (SD): neutral<negative<positive (all *p*<.05).

### The influence of depression, anxiety, and gender

**Figure 2:**
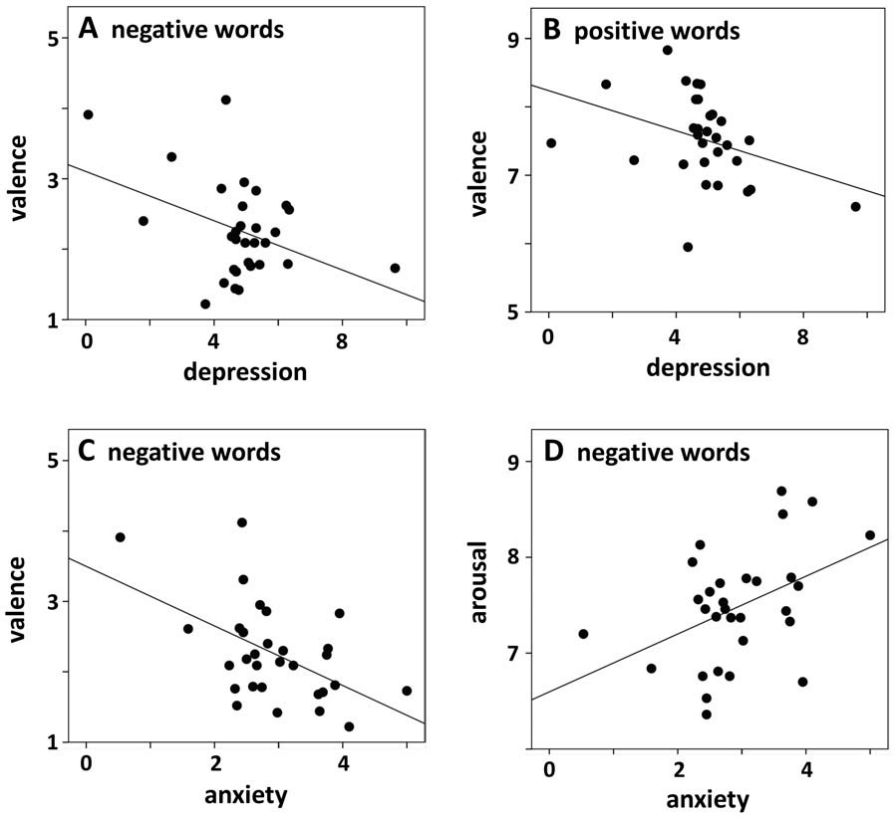
Scatter plots for depression scores and mean valence ratings for negative (A) and positive words (B), and for anxiety scores and mean valence (C) and arousal (D) ratings for negative words. Note that every data point represents a word in Figure 1, but a participant in Figure 2.

Mean depression, anxiety, and stress scores in the present participants resemble those found in representative samples (depression M = 4.83, SD = 1.57; anxiety M = 2.93, SD = 0.85; stress M = 7.07, SD = 2.92; [28], [29]). To test the influence of depression, anxiety, and stress on valence and arousal ratings we correlated the individual questionnaire scores with the mean ratings in each word group (e.g. mean valence rating of negative words). This yielded the correlational pattern reported in Table 2. It indicates that depression is related to more negative valence ratings for negative stimuli and similarly more negative, or less positive ratings for positive words (see Figure 2A and B). Depression did not correlate with arousal ratings and with valence ratings of neutral stimuli. Anxiety, in contrast, was related to more negative valence ratings and enhanced arousal ratings of negative stimuli (see Figure 2C and D), but no other significant correlations were observed. Stress did not correlate with any of the ratings.

**Table 2:** Correlations of depression, anxiety, and stress with the mean valence and arousal ratings of negative, neutral, and positive words.

		valence			arousal	
	negative	neutral	positive	negative	neutral	positive
depression	−.40**	−.14	−.37*	.14	.17	−.33
anxiety	−.52**	.17	.12	.43*	.11	−.02
Stress	.05	−.16	−.03	−.22	−.02	−.17

*<.05.

**<.01.

We also tested if the gender of the raters had an influence on the ratings and found a significant interaction of gender and valence ratings (see Table 1; *F*(2,56) = 4.4, *p*<.05) that indicated that female participants showed more negative ratings of negative words (*p*<.05) and a trend towards more positive ratings of positive words (*p*<.10). Valence ratings of neutral words did not differ. There was also no effect of gender on arousal ratings.

## Discussion

The present study had two major goals, (1) to provide a database of short auditory emotional recordings of German one-word utterances, and (2) to test the influence of the rating participants' current emotional state on affective norms. To this end, 480 recordings of emotional words taken from the LANG database on visual affective word norms were rated in valence and arousal. The data show very consistent rating results, yielding three subsets of highly arousing negative and positive stimuli as well as neutral words low in arousal. The quadratic relation of valence and arousal conforms to a large number of previous studies with different emotional material including pictures and visually presented words [30], [31]. It therefore supports the comparability of the present norms with previous data. In line with the visual ratings from the LANG and previous datasets on emotional speech [32], we used dimensional ratings in valence and arousal to characterize the stimuli. While valence and arousal explain the biggest proportion of variance in word ratings (for details see [33]), it seems pertinent to also use categorical emotion concepts for emotional speech [12]. Future studies could potentially combine both concepts to promote a fuller understanding of emotional speech perception (see e.g. [5]).

An important characteristic of the present database is that the semantic and prosodic information are congruent regarding their affective meaning. It has been shown that auditory stimuli whose semantic and prosodic emotional valence are not identical (e.g. neutral semantic meaning spoken in a happy tone of voice) are perceived as incongruent and are processed differently than congruent stimuli [34]. We avoided this ambiguity for the present database to enable use of the stimuli in studies that investigate the attentional, memory, and other effects of auditory verbal emotion. Future databases could also consider including incompatible stimuli, which could help to disentangle the specific contributions of semantic and prosodic information.

We also tested the influence of depression and anxiety on valence and arousal ratings. The more negative ratings of negative and positive stimuli by participants scoring high in depression as well as the increased arousal ratings of negative stimuli by participants scoring high in anxiety correspond well with neuroimaging data that showed increased responding of the amygdala and other limbic regions to emotional stimuli in clinical and subclinical anxiety and depression [14], [15], [16], [17], [18], [19]. This result makes it necessary for studies that select stimuli from databases to check if the affective norms apply to their participant samples. This also requires future databases to report the depression and anxiety scores of their rating samples, which should ideally be in the normal range of the population as was the case in the present study. Because we tested healthy participants, the range of depression and anxiety scores was relatively small. To further validate the correlations with valence and arousal ratings observed here, future studies should test more diverse populations, also including patients with depression or anxiety. Interestingly, there were no significant correlations of valence and arousal ratings with the stress scale. Here, one might have expected a relation as stress has been shown to increase amygdala responses to emotional stimuli [35] and also to slow performance when emotional distracters are presented [36]. However, most previous studies looked at acute stress, while the present questionnaire rather assesses chronic stress levels. It would be interesting to also test the influence of acute stress on the conscious evaluation of emotional stimuli in valence and arousal ratings in future studies.

In addition to these interindividual differences, ratings also differed for male and female raters. Women rated negative stimuli more negative than men and also showed a tendency towards more positive ratings of positive stimuli. This is in line with previous reports of more accurate recognition of emotional vocalizations [5], [37] and also corresponds to accelerated recognition and enhanced cortical responses to emotional speech in women [3], [38].

Another interesting question concerns the relation of the present auditory norms to the previously acquired visual norms. This relation has been statistically examined and is reported in a previous publication (see [39]). As expected, visual and auditory ratings were highly correlated, thus providing cross-modal validation of the affective norms contained in the visual and auditory databases.

There were two meaningful differences between the word groups in the present study. Positive words were rated lower in arousal than negative words and were longer in duration than both negative and neutral words. This was the case even though there were no differences in the ratings of the visual stimuli or the number of letters and syllables (see LANG norms [13]). Even though significant, these differences were very small. Nevertheless, future studies that require tight control of these factors should carefully select the stimuli from the present database, not using the entire groups of words, but matching stimuli from the different word groups.

One of the objectives of the present study was to create stimuli that can potentially be used in a wide variety of cognitive experimental experiments, which usually require short duration stimuli. As the mean duration of the current stimuli is approximately 670 ms (ranging 350 from to 1450 ms), this criterion is met. Researchers can select stimuli varying in valence and arousal from the database and can control for such timing issues. In this regard, the stimuli in the present database have already been successfully used in an electroencephalography and functional magnetic resonance imaging study on the influence of emotional speech on cognitive control [40], [41], which demonstrates their potential for use in experimental paradigms requiring short duration stimuli. Furthermore, the stimuli could be used in clinical samples to elucidate deficits in the processing of emotional speech, for example in Parkinson's Disease [42], [43], Bipolar Disorder, or Schizophrenia [44]. Thus, we hope the present database will fuel future research on emotional speech perception, in particular, investigations on the impact of emotional speech on cognitive functioning and their neural underpinnings.

## Supporting Information

Supporting Information S1 **LANG-audition database.** The database includes auditory recordings of each word stimulus and a table with normative data and additional information on each word (English translation, frequency, number of letters and syllables, visual ratings in valence, arousal, and concreteness, auditory ratings in valence and arousal, and the duration, mean, minimum, and maximum pitch, and pitch variation).
(RAR)
